# Symptomatic Carotid Artery Stenosis: Surgery, Stenting, or Medical Therapy?

**DOI:** 10.1007/s11936-017-0564-0

**Published:** 2017-07-05

**Authors:** Ashley M. Wabnitz, Tanya N. Turan

**Affiliations:** Division of Neurology, Medical University of South Carolina, 19 Hagood Ave, Harborview Office Tower Suite 501, Charleston, SC 29425-8050 USA

**Keywords:** Carotid atherosclerosis, Symptomatic carotid disease, Carotid stenosis, Cerebrovascular disease, Intracranial atherosclerosis

## Abstract

Symptomatic carotid artery disease is a significant cause of ischemic stroke, and these patients are at high risk for recurrent vascular events. Patients with symptoms of stroke or transient ischemic attack attributable to a significantly stenotic vessel (70–99% luminal narrowing) should be treated with intensive medical therapy. Intensive medical therapy is a combination of pharmacologic and lifestyle interventions consistent with best-known practices as follows: initiation of antiplatelet agent or anticoagulation if medically indicated, high potency statin medication, blood pressure control with goal blood pressure of greater than 140/90, Mediterranean-style diet, exercise, and smoking cessation. Further, patients who have extracranial culprit lesions should be considered for revascularization with either carotid endarterectomy or carotid angioplasty and stenting depending on several factors including the patient’s anatomy, age, gender, and procedural risk. Based on current evidence, patients with symptomatic intracranial stenosis should be managed with intensive medical therapy, including the use of dual antiplatelet therapy with aspirin and clopidogrel for the first 90 days following the ischemic event. While the literature has shown a stronger benefit of revascularization of extracranial symptomatic disease among certain subgroups of patients with greater than 70% stenosis, there is less benefit from revascularization with endarterectomy in patients with moderate stenosis of 50–69% if the surgeon’s risk of perioperative stroke or death rate is greater than 6%.

## Introduction

Carotid artery stenosis is a major contributor to ischemic stroke, found to be responsible for 15–20% of all strokes. Appropriate medical and/or revascularization management of these patients may provide a significant reduction in stroke burden in the future, particularly as the number of those affected by carotid artery atherosclerosis is likely to increase as rates of type 2 diabetes, hypertension, and obesity continue to rise.

Symptomatic carotid stenosis is commonly defined as stenosis in the internal carotid artery, either intracranial or extracranial, leading to symptoms of amaurosis fugax, transient ischemic attacks, or ischemic stroke ipsilateral to the lesion. Degree of stenosis varies among the major therapeutic trials studying treatment of carotid stenosis, but severe stenosis (70–99%) has been demonstrated to confer the highest risk for recurrent stroke or TIA. Current treatment options for symptomatic carotid stenosis vary based on location of the stenosis - extracranial vs. intracranial.

### Extracranial symptomatic carotid stenosis

Extracranial carotid atherosclerosis is typically seen at the carotid bifurcation extending to the intracranial internal carotid artery in a portion referred to as the cervical segment. Extensive evidence, including several major randomized control trials, has shown that patients with severe symptomatic extracranial atherosclerosis benefit from revascularization in addition to medical management [1–3]. Revascularization techniques for extracranial carotid atherosclerosis include carotid endarterectomy (CEA) or carotid angioplasty and stenting (CAS). Revascularization is discussed in more details below.

### Intracranial symptomatic carotid stenosis

The intracranial carotid artery begins at the petrous segment and continues in the direction of blood flow to the lacerum, cavernous, clinoid, ophthalmic, and communicating segments where it ends at the carotid terminus. While extracranial disease is best managed with a combination of intensive management of vascular risk factors and revascularization, intensive medical management has been demonstrated to be superior to revascularization in patients with severe (70–99%) intracranial stenosis. Revascularization techniques in this population are limited to angioplasty and stenting given limited accessibility in the skull base. Further details of medical management and CAS in this population are discussed below.

## Medical treatment

In all patients with severe symptomatic carotid stenosis, aggressive medical management should include a combination of antiplatelet therapy, high potency statin, blood pressure control, and lifestyle risk factor modification as outlined below. Recent large clinical trials of patients with carotid stenosis have reported that even in the age of modern medical management, less than 20% of patients had optimal risk factor control at baseline, suggesting that the importance of risk factor control remains overlooked, even in patients with known atherosclerotic disease [4•].

### Lifestyle modification: diet, exercise, and smoking cessation

#### Diet

Previously published guidelines for stroke prevention have recommended the DASH diet with a focus on vegetables, fruits, whole grains, low-fat dairy products, poultry, fish, legumes and nuts, vegetable oils and limited amounts of red meat, and sugar and sugar-sweetened beverages, for reducing risk of stroke and also showing efficacy in reducing blood pressure and cholesterol [5, 6]. New evidence has emerged that the Mediterranean diet, which is similarly focused on fruit, vegetable, fish, and whole grains but allows for the inclusion of low-fat dairy products, olive oil, and moderate alcohol intake, is beneficial in reducing stroke risk and cardiovascular disease [7]. Both diets encourage low intake of sugars, and red meat with olive oil instead of vegetable oil and moderate amounts of alcohol, primarily wine, being recommended as beneficial at reducing strokes. A study looking at stroke classification and pre-morbid Mediterranean diet adherence showed that patients with a low adherence to the Mediterranean diet were more likely to have large artery atherosclerosis as the etiology for their stroke [8].

#### Exercise

Current recommendations from the AHA/ACC are for adults to participate in moderate- to vigorous-intensity activity lasting on average 40 min three to four times per week to reduce the risk of cardiovascular disease [6]. In a multivariate analysis of patients with severe intracranial atherosclerotic stenosis, physical inactivity was shown to increase the likelihood of recurrent stroke, heart attack, or vascular death by fivefold [9].

#### Smoking cessation

Smoking has been widely demonstrated to contribute to first-time stroke as well as cardiovascular disease in general, and smoking cessation is recommended for prevention of further vascular events [5, 10]. Some symptomatic carotid revascularization trials have shown evidence of smoking status as independent risk factor for future stroke, heart attack, or death [11].

### Pharmacologic treatment

#### Antihypertensives

Patients who have had prior stroke or TIA attributed to carotid stenosis should be initiated or continued on blood pressure-lowering therapy when blood pressure exceeds 140/90 [5]. Several studies have now shown that the prior common practice of allowing chronic permissive hypertension to increase cerebral perfusion among patients with extracranial or intracranial carotid artery stenosis increases the risk of recurrent stroke, even among those with high-grade stenosis or documented perfusion deficits [9, 12, 13]. The recently published SPRINT trial supports intensive blood pressure control in patients without prior stroke or diabetes with a goal of systolic blood pressure <120 mmHg, as this was shown to significantly reduce the rates of significant cardiovascular events in this non-stroke population [14]. However, the benefit of a target SBP < 120 over <140 among patients with symptomatic carotid disease has not been systematically studied.

#### Statins

Aggressive lowering of low-density lipoprotein cholesterol (LDL-C) with high-potency statin therapy (e.g., atorvastatin 80 mg) reduces risk of further cardiovascular events in patients with prior stroke or TIA and is indicated in all patients with stroke or TIA who do not have contraindications according to several published guidelines [5, 15]. Additional lipid-lowering agents (e.g., ezetimibe or PCSK9 inhibitors) may also be added in cases where a specific LDL goal is sought. For example, treating to LDL-C < 100 mg/dL may be sufficient for most patients, but <70 mg/dL may be a more appropriate target for patients with diabetes or those otherwise considered very high-risk [16]. In addition to LDL-C lowering, there is evidence that use of statins slows the progression of carotid atherosclerosis [17]. A study of atorvastatin found a dose-response relationship, with atorvastatin 80 mg daily showing greatest benefit at stabilizing vulnerable plaques in patients with symptomatic carotid stenosis [18].

#### Antiplatelet agents

Antiplatelet therapy remains a cornerstone of stroke prevention in patients with cerebral atherosclerosis. The use of aspirin, clopidogrel, aspirin-dipyridamole, or ticagrelor in patients with symptomatic carotid stenosis is a standard treatment. However, few trials or subgroup analyses comment on the superiority of one antiplatelet agent over another for reducing recurrent stroke risk specifically among patients with symptomatic carotid stenosis, either extracranial or intracranial. One trial compared aspirin 325 mg to clopidogrel 75 mg daily in patients with recent non-cardioembolic stroke, including those with carotid stenosis, and showed no significant benefit of clopidogrel over aspirin for reducing risk of future stroke, myocardial infarction, or death within 1 year [19]. The PRoFESS study compared clopidogrel to aspirin-dipyridamole in patients with recent non-cardioembolic stroke and found the latter to be noninferior to clopidogrel for secondary prevention of ischemic stroke [20]. In a recent pre-specified subgroup analysis of the SOCRATES trial, ticagrelor was shown to be superior to aspirin for preventing early recurrent vascular events at 90 days among patients with recent stroke or TIA attributed to ipsilateral atherosclerotic stenosis [21•].

Dual antiplatelet therapy (DAPT) with aspirin 325 mg and clopidogrel 75 mg for a short duration may be beneficial for stroke prevention in patients with large artery atherosclerosis. Patients with severe intracranial atherosclerosis randomized to the aggressive medical management arm of the SAMMPRIS trial who were given dual antiplatelet therapy for 90 days had lower rates of recurrent vascular events than similar patients using aspirin or warfarin in a previous trial [22•, 23]. Similarly, short-term DAPT for 7 days was compared to aspirin alone and found to reduce microembolus signals detected by transcranial Doppler (TCD) and clinical ischemic events in patients with symptomatic extracranial and intracranial carotid stenosis in the CARESSS and CLAIR trials [24, 25]. Therefore, short-term DAPT has become widely used for stroke prevention among patients with recently symptomatic carotid stenosis. However, DAPT for longer than 90 days is not recommended for stroke prevention due to the increased risk of major bleeding, which outweighs any potential additional benefit in stroke risk reduction [26, 27].

### Surgical procedures

#### Carotid endarterectomy

CEA is a surgical procedure wherein direct revascularization is achieved by opening the lumen and removing the atherosclerotic plaque in an extracranial internal carotid artery. Three landmark studies reported in the 1990s, the European Carotid Surgery Trial (ECST), the North American Symptomatic Carotid Endarterectomy Trial (NASCET), and the Veterans Affairs Cooperative Studies (VACSP), showed benefit of CEA compared to medical management for preventing future vascular events, particularly in patients with non-occlusive high-grade atherosclerotic stenosis measuring >70% luminal narrowing [1–3]. The substantial benefit of CEA for stroke prevention was best demonstrated in the North American Symptomatic Carotid Endarterectomy Trial (NASCET) where the number needed to treat (NNT) in order to prevent one major stroke at 2-year follow-up was 6 [1]. For patients with moderate stenosis of 50–69%, the benefit from CEA was less robust, with a number needed to treat of 15 to prevent one ipsilateral stroke [1]. The most recent guidelines encourage pursuing CEA for patients with moderate stenosis only if the surgeon performing the endarterectomy has a perioperative stroke or death rate of <6% [5]. This is based on the published perioperative stroke or death rate of between 6% in more recent studies and 6.7% in NASCET [1, 28]. However, given that both medical therapy and revascularization procedures have made significant advances since that time with lower event rates seen in population-based studies of both therapies, several experts have called for new randomized trials to reassess this clinical question, particularly among subsets of patients who were previously shown to benefit less from CEA [29, 30]. These subgroups have been identified in several analyses, including a pooled analysis of patients with greater than 50% stenosis from prior CEA trials. Table 1 shows the NNT to prevent one ipsilateral ischemic stroke in select subgroups within 5 years based on the pooled data from NASCET and ECST [31].

**Table 1:** Number needed to treat (NNT) with carotid endarterectomy to prevent one ischemic stroke in 5 years based on pooled data from NASCET and ECST

Subgroup	NNT
Men	9
Women	36
Age < 65 years old	18
Age > 75 years old	5
Time from last event <2 weeks	5
Time from last event >12 weeks	125

##### Women

The evidence for sex differences in morbidity and mortality from CEA has been mixed. NASCET suggested that the periprocedural morbidity and mortality rate was higher for women, resulting in less benefit from CEA. However, subgroup analyses of CREST and other more recent population-based observational studies have failed to show a difference in surgical morbidity or mortality for carotid endarterectomy by sex [32, 33]. The lack of sex as a risk modifier in more recent studies is likely multifactorial but may be due to improved best medical management since the initial major clinical trials or better representation of women in more recent studies.

##### Delayed endarterectomy

Timing of endarterectomy is recommended within 2 to 14 days following an ischemic event [5]. This is based on evidence that with medical management alone, the greatest stroke risk is in the first 2 weeks and subsequently tapers off, with rates being similar to asymptomatic carotid stenosis at 2–3 years [31, 34–36]. More recent retrospective studies looking at early (within 2 weeks) versus delayed endarterectomy for symptomatic carotid disease >50% have shown no increased risk of morbidity and mortality with early CEA, which is consistent with a post hoc analysis of the CREST data set [37–40] that also showed that early CEA is safe. A concern of early revascularization is hemorrhagic transformation due to cerebral hyperperfusion syndrome wherein restored blood flow to the area of friable ischemic tissue is thought to increase the risk of hemorrhage into the stroke bed. However, a case series published in 2016 by Azzini et al. found that among 34 patients who underwent surgery for severe carotid stenosis 12 h–2 weeks after an ischemic event and received IV thrombolysis, none had hemorrhagic transformation [41]. Similarly, another retrospective review of 761 symptomatic patients with moderate-to-severe carotid stenosis who underwent CEA reported only two patients with resulting intracerebral hemorrhage on follow-up imaging, and only one of those was attributed to hyperperfusion syndrome [39, 41].

##### Retinal ischemia

Transient monocular partial or complete vision loss, also referred to as amaurosis fugax, is a common symptom of retinal ischemia related to the internal carotid artery. Future ischemic stroke risk in these patients is much lower than those with cerebral ischemia seen in other types of transient ischemic attacks [42, 43]. A study of patients in the NASCET trial with high-grade (70–99%) carotid stenosis found that patients with retinal ischemia had a 2-year ipsilateral ischemic stroke risk of 16.6% compared to a 43.5% risk in those with hemispheric symptoms (*p* = 0.002) [42]. In addition, a subgroup analysis found that only high-risk patients (defined as three or more of the following: age >75, male sex, history of hemispheric TIA or stroke, history of claudication, stenosis of 80 to 94%, and absent collateral circulation) would have benefit for CEA with a number needed to treat of 7 to prevent one ipsilateral stroke over the 3-year period compared to the medically managed group. For moderate-risk patients including those who presented with amaurosis fugax, the NNT was 20, indicating less benefit to pursuing carotid endarterectomy [43]. Importantly, this data was from the NASCET trial, and since that time, advances in medical management including the standard use of statin therapy may make the incremental benefit from CEA even less advantageous in the subset of patients with retinal ischemia.

### Interventional procedures

#### Carotid angioplasty and stenting

CAS is an endovascular revascularization procedure performed utilizing balloon angioplasty and deployment of a stent over the culprit plaque to increase the size of the lumen and reduce recurrent stroke risk. Several randomized controlled trials evaluating stenting vs. endarterectomy for extracranial carotid stenosis have been completed: the Carotid Revascularization Endarterectomy vs. Stenting Trial (CREST), Stenting and Angioplasty with Protection in Patients at High Risk for Endarterectomy (SAPPHIRE), Endarterectomy Versus Angioplasty in Symptomatic Severe Carotid Stenosis (EVA-3S), and the International Carotid Stenting Study (ICSS). These trials have shown comparable long-term results of CAS to CEA at preventing disabling or fatal strokes after the periprocedural period [44–47]. Some patient populations may benefit from one procedure over the other, as outlined below. Typically, patients who are felt to be particularly favorable for CAS are those who are high risk for surgery, such as those with contralateral occlusion, anatomical variations making surgical access technically difficult (radiation injury, history of prior neck dissection, presence of tracheostomy), or those with severe medical comorbidities making them high-risk for open surgery [48]. Typically, insurance coverage for CAS outside of a clinical trial is currently limited to those with symptomatic >70% carotid stenosis who are thought to be at high risk of complications from CEA [49].

Many of the randomized trials comparing CEA and CAS report a higher periprocedural stroke rate in CAS patients and/or a higher MI rate in CEA patients [44, 45]. The recently reported 10-year follow-up of CREST patients demonstrated no differences between CAS and CEA in the long-term risk of stroke, MI, or vascular death (11.1 vs. 9.9%, *p* = 0.51) [50•]. However, some argue that the rigorous selection criteria for endovascular surgeons in the randomized control trials result in a lack of generalizability to endovascular surgeons in practice, and therefore, there may be a greater risk from CAS done in a community setting. For example, a recently published retrospective analysis of Medicare beneficiaries who underwent revascularization showed that the Medicare patients in practice had a periprocedural mortality rate of 1.7%, which was more than double the mortality rate in CREST and SAPPHIRE (0.7 and 0.6%, respectively) [51]. The higher mortality rate was attributed the fact that community physicians performing CAS would likely not meet the highly selective credentialing requirements (high procedure volumes and low periprocedural complication rates) applied to the physicians in the RCTs [44, 45, 51]. The highest risk of morbidity and mortality was in the more elderly patients with higher surgical risk and symptomatic carotid lesions [51]. Another retrospective database review of patients admitted to the hospital for CAS or CEA with both symptomatic and asymptomatic carotid stenosis showed similar significantly increased periprocedural morbidity and mortality associated with CAS compared to CEA for symptomatic carotid stenosis patients [52].

Timing of angioplasty and stenting has also been controversial due to concern for cerebral hyperperfusion syndrome. Recent studies have compared morbidity and mortality following early CAS compared to delayed CAS. Liu et al. studied 63 patients with severe stenosis undergoing CAS at less than 1 week compared to 57 patients undergoing delayed intervention at 1 month and found improved functional outcomes and no increased rate of second stroke, MI, or death in the early CAS group [53]. In contrast, Song et al. reported a retrospective analysis of 206 patients undergoing CAS for moderate-to-severe stenosis that found a significantly higher 30-day event rate of ipsilateral stroke or death of 12.8% in the early CAS group (within 14 days) compared to just 0.8% in the delayed CAS group (mean timing of CAS was 52.6 ± 36.94 days) [54]. This finding did not extend beyond the 30-day period (31 days to 1 year) follow-up, wherein there was no significant difference between groups [54]. A CREST post hoc analysis did not show any relationship between timing and significant adverse events in the 583 patients in the CAS group [40]. Given the known increased risk for second stroke occurring within the first 2 weeks, the recommendation remains to pursue early revascularization within 14 days of ischemic symptoms.

Age > 70: a meta-analysis of three major trials comparing CEA to CAS in patients with symptomatic stenosis suggests that CEA is favorable in patients >70 years, with a rate of stroke or death at 120 days after randomization of 12% for CAS vs. 5.9% in those undergoing CEA [55]. Pooled population analyses of patients with symptomatic carotid stenosis obtained from randomized controlled trials within the Carotid Stenosis Trialists’ Collaboration have corroborated this finding, also showing a significant increase in periprocedural stroke and death risk of approximately 11% in patients greater than 70 years old undergoing CAS, reinforcing prior recommendations for CEA over CAS in this population [56]. Furthermore, the International Carotid Stenting Study also showed increasing age to be independently associated with increased risk of MI, death, or stroke within 30 days of CAS. After the periprocedural period, there does not seem to be a persistent increased risk of stroke in older patients undergoing revascularization, which may mean that improvements in CAS procedures, such as better embolic protection devices, may decrease the relative superiority of CEA.

##### Intracranial carotid stenosis

Two major multicenter randomized clinical trials comparing revascularization and medical therapy have been completed in patients with severe (>70%) intracranial stenosis—Stenting and Aggressive Medical Management for Preventing Recurrent Stroke in Intracranial Stenosis (SAMMPRIS) and Vitesse Intracranial Stent Study for Ischemic Stroke (VISSIT) [57, 58]. SAMMPRIS was stopped early due to the clear benefit of intensive medical therapy alone over revascularization plus intensive medical therapy for prevention of recurrent stroke, and VISSIT showed similar benefit overall. Approximately 20% of patients in the SAMMPRIS trial had intracranial internal carotid artery (ICA) stenosis. ICA patients in the medical arm had a lower rate of the primary endpoint (recurrent stroke in the territory of the stenotic vessel, intracranial hemorrhage, or death) compared to the stenting arm during mean follow-up of 32.4 months (14.9 vs. 23.9%, *p* = 0.0193) [22•]. In the VISSIT trial, overall, there was also a lower rate of the primary endpoint (recurrent stroke or hard TIA in the territory of the stenotic vessel) at 1 year in patients in the medical management group compared to those in the stenting group (9.4% vs. 34.5%, *p* = 0.003), but the event rates specific to ICA patients were not reported [58]. For patients with severe symptomatic intracranial stenosis, there is no benefit to angioplasty and stenting over aggressive medical management alone [22•, 58].
